# Exploring functional data analysis and wavelet principal component analysis on ecstasy (MDMA) wastewater data

**DOI:** 10.1186/s12874-016-0179-2

**Published:** 2016-07-12

**Authors:** Stefania Salvatore, Jørgen G. Bramness, Jo Røislien

**Affiliations:** Norwegian Centre for Addiction Research, University of Oslo, Oslo, Norway; Oslo Centre for Biostatistics and Epidemiology, Institute of Basic Medical Sciences, Oslo, Norway

**Keywords:** Wastewater-based epidemiology, Stimulant drugs, Functional principal component analysis, Wavelet PCA

## Abstract

**Background:**

Wastewater-based epidemiology (WBE) is a novel approach in drug use epidemiology which aims to monitor the extent of use of various drugs in a community. In this study, we investigate functional principal component analysis (FPCA) as a tool for analysing WBE data and compare it to traditional principal component analysis (PCA) and to wavelet principal component analysis (WPCA) which is more flexible temporally.

**Methods:**

We analysed temporal wastewater data from 42 European cities collected daily over one week in March 2013. The main temporal features of ecstasy (MDMA) were extracted using FPCA using both Fourier and B-spline basis functions with three different smoothing parameters, along with PCA and WPCA with different mother wavelets and shrinkage rules. The stability of FPCA was explored through bootstrapping and analysis of sensitivity to missing data.

**Results:**

The first three principal components (PCs), functional principal components (FPCs) and wavelet principal components (WPCs) explained 87.5-99.6 % of the temporal variation between cities, depending on the choice of basis and smoothing. The extracted temporal features from PCA, FPCA and WPCA were consistent. FPCA using Fourier basis and common-optimal smoothing was the most stable and least sensitive to missing data.

**Conclusion:**

FPCA is a flexible and analytically tractable method for analysing temporal changes in wastewater data, and is robust to missing data. WPCA did not reveal any rapid temporal changes in the data not captured by FPCA. Overall the results suggest FPCA with Fourier basis functions and common-optimal smoothing parameter as the most accurate approach when analysing WBE data.

**Electronic supplementary material:**

The online version of this article (doi:10.1186/s12874-016-0179-2) contains supplementary material, which is available to authorized users.

## Background

Ecstasy (MDMA), along with cocaine, amphetamine and methamphetamine, are central nervous system stimulants that cause euphoria with feelings of increased confidence, sociability and energy, making them popular drugs of abuse [1, 2]. However, stimulant use has numerous negative effects, such as insomnia, anxiety, mood disturbance, violent behaviour and dependence, making them a public health concern [3–5]. MDMA is not the most prevalently used illicit drug in Europe [6, 7], but its high weekend use compared to weekday use [8] has been a source of concern.

Traditionally, estimates of the consumption of illicit drugs have been derived from population-based surveys and administrative databases such as medical records, crime statistics, drug production and seizure data [6]. Population-based surveys are, however, often characterized by low response rates because of sensitive questions [9], while the use of administrative databases presents several methodological challenges since any analysis targets selected populations [10–12]. Data gathered from treatment facilities and drug-related programmes can underestimate prevalence as the number of places in treatment tend to be limited [10], while drug-related offences may overestimate prevalence [11, 12].

Wastewater-based epidemiology (WBE) is a novel approach in drug use epidemiology. The concentration of illicit drugs in wastewater is measured directly, thus overcoming some of the problems related to surveys. WBE has shown promising results at local, national and international levels [8, 13, 14], and naïve statistical analyses of wastewater data have demonstrated differences between concentrations of drugs detected in wastewater on weekdays and at weekends [15–17].

Recently, functional principal component analysis (FPCA) has been explored as a statistical method for analysing wastewater data [18]. The approach was found not only to be well suited for extracting useful information about the different drug loads during the course of a week, but also extracted detailed information that would otherwise be lost when using more traditional statistical methods. It can easily be argued that functional data analysis (FDA) is a reasonable approach to analysing temporal wastewater data [18], but there is a concern that the basis functions of the FDA framework might be too smooth to model the rapid temporal changes in drug load curves that can occur over the course of a week, especially the change between weekdays and weekend. Alternative, more flexible, statistical approaches should also be explored.

Wavelets have a long tradition in time series analysis [19]. Wavelet basis functions are localized in both frequency and time domains simultaneously, allowing for the extraction of features that are less smooth from temporal data [20, 21]. Wavelet-based principal component analysis (WPCA) has recently been applied successfully to analysis of foetal movement monitoring data [22, 23]. The temporally more flexible WPCA could be able to detect rapid temporal changes in wastewater data.

The aim of this study was to explore whether the well-established FDA framework is sufficiently flexible to model temporal changes properly in wastewater data. We compared the stability of results, applying traditional principal component analysis (PCA) [24], FPCA [25, 26], and WPCA [22, 23] using various basis functions and smoothing approaches, and investigated the sensitivity to missing data.

## Methods

### Data material

Raw sewage samples were collected from the inlet of 47 sewage treatment plants in 42 cities from 21 European countries, servicing a combined population of approximately 24.7 million inhabitants [27]. Samples were collected from each location over seven consecutive days, starting for 36 of the 42 cities on Wednesday 6th March 2013 and ending on Tuesday 12th March 2013. For the remaining six cities sampling during this week was not possible, and a different week in the same month was chosen. At all locations, automated sampling devices were used to collect subsamples over 24 h. These subsamples were then pooled to a 24 h composite sample. For cities with more than one sewage treatment plant, results were combined to a city average using a weighted mean [27]. Daily mass loads normalized by the population size of the catchment (mg/10 000 people/day) were considered. Four cities had no values above the limit of quantification (LOQ) and were thus excluded, leaving a total of 38 cities for further statistical analysis.

### Statistical analysis

#### Data description

The unit of observation in the analysis is a seven day week starting Wednesday and ending Tuesday. As wavelet analysis generally requires individual time series to have a length of a power of two observations [21], we added the first observation to the end of the time series, generating an eight day time series, for ease of comparison. This additional day is needed only for technical purposes and does not have any impact on the results [21]. Missing data across all the 38 cities was 2.2 %. As standard frequentist functional data analysis (FDA) needs complete data sets for analysis, we performed single imputation [28] using the bootstrapping-based expectation maximization algorithm [29], before proceeding with the analysis on the imputed dataset. Moreover, the wastewater data was heavily skewed, and the data was log-transformed prior to further analysis.

#### Principal component analysis

Principal component analysis (PCA) is a statistical methodology which is used to reveal the internal structure of the data in order to explain variability [24]. Let *N* indicate the sets of observations, the core concept in PCA is that of taking a linear combination of the variable values within each set,

1 $$ {f}_i\kern0.5em =\kern0.5em {\varSigma}_{j\kern0.5em =\kern0.5em 1}^p\kern0.5em {\beta}_j{x}_{ij},i\kern0.5em =\kern0.5em 1,\kern0.5em \dots \kern0.5em ,\kern0.5em N, $$

where *β*_*j*_ is a weighting coefficient applied to the observed values *x*_*ij*_ of the *j*^*th*^ variable. In our data *p* = 8 (days) and *N* = 38 (cities). PCA implies identifying a sets of normalized weights that maximize variation in *f*_*i*_’s, where the greatest variance is explained by the first coordinate, that is, the first principal component (PC), and the second greatest variance on the second coordinate, and so on. The PCs are mutually uncorrelated by construction [24].

Using traditional PCA, each day of the week is considered a single variable and each PC resulting from the PCA is defined as a linear combination of the original variables. Since in PCA the load of a drug at a given day is assumed to be independent of the drug load at any other day, be it preceding or following days, the correlation between individual days is not taken into account. This assumption is however likely to be violated for wastewater data where consecutive days are naturally correlated in time. PCA on temporal data will yield temporal PCs, but as intra-correlation of individual time series is not modelled, the temporal aspect of the data will be ignored, leading to a lower ability to recover the true underlying signal of interest [30]. So while PCA is a well-known, well-established statistical method for extracting structure in the data, results should be interpreted with care for temporal data.

#### Functional principal component analysis

FDA is a statistical methodology specifically developed for analysing temporal data [25]. The first step of FDA is to fit a mathematical function to the temporal observations, and the statistical analysis is then performed on this mathematical function rather than the raw data. The time series for the 38 European cities were converted into 38 continuous smooth curves using both Fourier and B-spline basis functions. The optimal smoothing was found using the generalized cross validation (GCV) criterion [31]. A single choice of smoothing parameter for all cities is usually recommended [32], but for exploratory purposes we also fitted an optimal individual smoothing parameter for each city. This smoothing removes the random day-to-day variation, e.g. non-systematic error, measurement error and normal fluctuations in the drug load.

Functional principal component analysis (FPCA) is an extension of traditional PCA to functional data [25]. We applied FPCA to identify the main temporal features across the individual 38 fitted smooth curves. In the functional context [25], where individual daily observations *x*_*i*_ are replaced with smooth functions *x*_*i*_(*s*), the discrete index *j* of the multivariate analysis (eq. 1) is replaced by the continuous index *s*, and the weights *β*_*j*_ become functions *β*_*j*_(*s*). Performing PCA in the functional context then becomes taking a linear combination of functions,

2 $$ {f}_i\kern0.5em =\kern0.5em \int \kern0.5em \beta (s)\kern0.5em {x}_i(s)ds,\kern0.5em i\kern0.5em =\kern0.5em 1,\kern0.5em \dots \kern0.5em ,\kern0.5em N, $$

where the summation over *j* in the traditional PCA (eq. 1) is replaced by integrations over *s* (eq. 2). As in traditional PCA, FPCA implies identifying a sets of normalized weighting functions that maximize variation in *f*_*i*_’s. These mutually independent functional principal component (FPC) curves explain the main modes of temporal variability across the individual fitted curves for all cities. Also, a score for each individual time series is provided, indicating the intensity with which each of the FPC patterns is present in that particular temporal ecstasy (MDMA) load curve. FPCA is performed on continuous functions *f*(*t*) instead of the original data points and the correlation between individual time points, here days of the week, is modelled through the fitted basis function for each city. The various types of basis functions applicable in the FDA framework gives flexibility in the types of temporal signals that can be modelled, given that the temporal behaviour of the data under study is sufficiently smooth.

#### Wavelet-based principal component analysis

Wavelets is a mathematical framework developed for analysing high-dimensional data, such as time series or images [19]. While FDA uses global basis functions, such as trigonometric functions, wavelet basis functions are localized in both time and space, allowing for modelling of less smooth temporal data, even spikes [20, 21]. Wavelet basis functions are generally not expressed explicitly as functions. Instead individual basis functions are specified by recursive difference equations conditioned on a mother wavelet [33]. The mother wavelet *Ψ*(*t*) and a corresponding father wavelet *φ*(*t*) can be interpreted as a high-pass and low-pass filter of the original data respectively.

The wavelet decomposition of a function *y*(*t*) is a projection of that function onto a wavelet basis as

3 $$ y(t)\kern0.5em =\kern0.5em {\varSigma}_{k\kern0.5em \in Z\kern0.5em cj0,k\kern0.5em \varphi j0,k}(t)\kern0.5em +\kern0.5em {\varSigma}_{j\kern0.5em =\kern0.5em j0}^{\infty}\kern0.5em {\varSigma}_{k\in Z\kern0.5em dj,k\kern0.5em \varPsi \mathrm{j},\mathrm{k}}\left(\mathrm{t}\right), $$

with *Z* the set of all integers, and *φ*_*j*0,*k*_(*t*) and *Ψ*_j,k_(t) the basis functions [21]. The scalars *c*_*j*0,*k*_(*t*) and *d*_*j*,*k*_(*t*), which can be combined into a long coefficient vector *B*_*i*_, then represent individual *i*’s temporal observations *y*_*i*_(*t*) in wavelet coefficient space.

Before proceeding with the wavelet principal component analysis (WPCA), for each vector of wavelet coefficients we applied wavelet shrinkage on the coefficients *B*_*i*_ to filter out the noise inherited from *y*_*i*_(*t*) [34]. Numerous thresholding rules exist. In this study, we considered universal thresholding [35] and Bayesian [36] wavelet shrinkage.

Wavelet-based principal component analysis (WPCA) is an application of standard PCA to the wavelet domain [22]. Performing PCA on the smoothed *B*_*i*_ coefficients in the wavelet domain using eq. 1, results in a set of new variables which are linear combinations of the smoothed wavelet coefficients *B*_*i*_. PCA in wavelet domain as applied here thus assumes independency of the coefficients *B*_*i*_. Intra-correlation of the individual time series is taken care of during the transformation process from time domain to wavelet domain (eq. 3). Back transforming the PCs obtained in wavelet domain to time domain gives the wavelet principal components (WPCs). The process also provides a score for each individual *y*_*i*_(*t*) indicating the intensity with which each of the WPC patterns is present in that particular temporal MDMA load curve. While WPCA is temporally more flexible than FPCA, it is also technically less tractable.

#### Bootstrapping

To compare analytical results from the different FPCA approaches, we constructed confidence intervals (CIs) using non-parametric bootstrapping [37, 38]. We performed FPCA on 1000 re-samples obtained by a random sample with repetition from the original 38 temporal data sets, calculating the pointwise empirical 95 % CI for each FPC. The procedure was repeated for both Fourier and B-spline basis functions, as well as no smoothing, common-optimal smoothing and individual-optimal smoothing parameters. Similar analyses were also performed for WPCA.

#### Sensitivity to missing data

In order to evaluate the robustness of FPCA to missing values we performed the FPCA for Fourier and B-spline basis functions and for the three different smoothing parameters, deleting at random 5, 10, 15 and 20 % of the original observations. Similar analyses were also performed for WPCA.

#### Values below LOQ

In wastewater-based epidemiology (WBE) the common practice of handling values below the LOQ is to replace those values with LOQ/2 [27]. This approach will, however, replace missing information with an identical value, that is, it will replace uncertainty with extreme precision [39]. We explored the consequence of this approach by comparing it to a more rigorous statistical procedure, namely replacing values below LOQ by random draws from a uniform distribution on the interval [0, LOQ].

#### Software

All analyses were performed in R 3.2.2 [40]. The imputation was performed using Amelia II and the *amelia* package [41] and the FPCA using package *fda* [26]. No R package for WPCA currently exists, and WPCA was performed by building on features of package *wavethresh* [21].

## Results

**Fig. 1:**
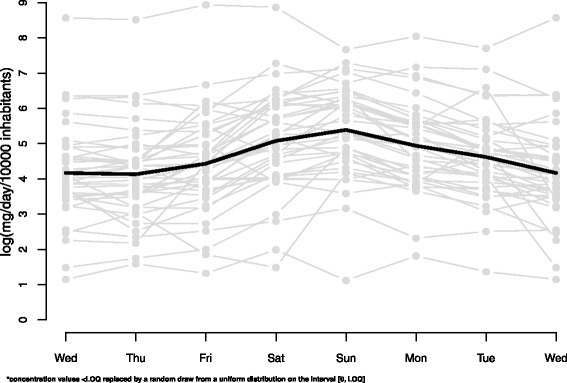
Raw data

The original data for each city load of ecstasy (MDMA) throughout the week, along with the day-by-day average, is shown in Fig. 1. The data indicate a slight increase in the drug load at the weekend.

### Principal component analysis

**Fig. 2:**
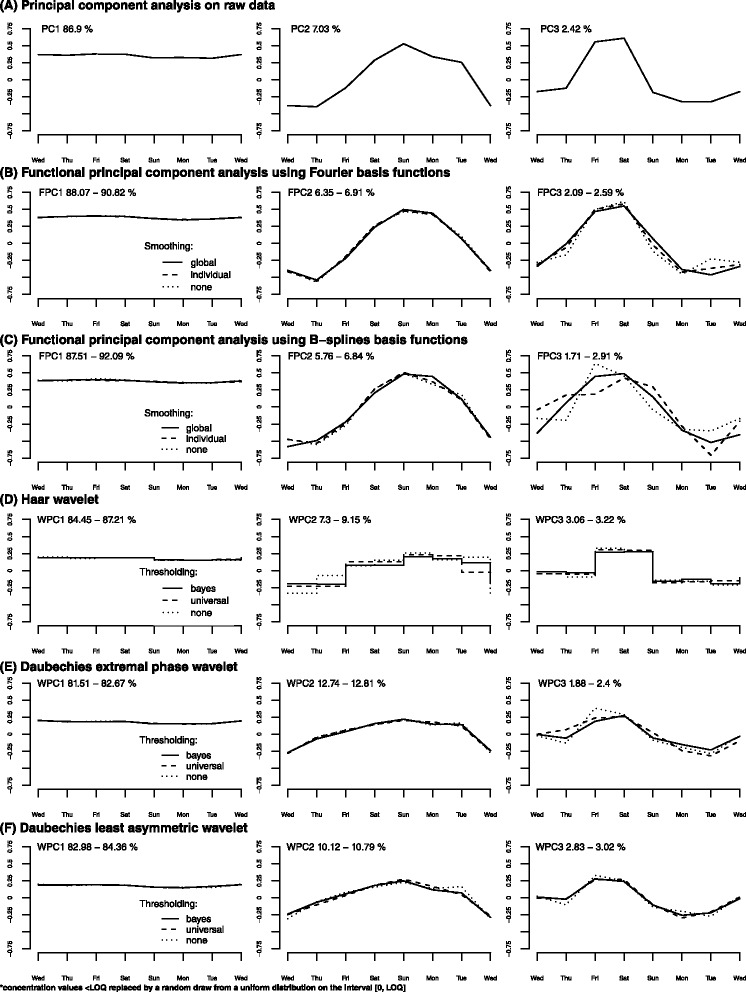
Principal component analysis (PCA), functional principal component analysis (FPCA) and wavelet-based principal component analysis (WPCA). Panel **a** – Principal components (PCs) resulting from a PCA on raw data; Panel **b** – Functional principal components (FPCs) resulting from a FPCA using Fourier basis functions and three different smoothing parameters; Panel **c** – Functional principal components (FPCs) resulting from a FPCA using B-splines basis functions and three different smoothing parameters. Panel **d**– Wavelet principal components (WPCs) resulting from a WPCA using the Haar mother wavelet and three different shrinkage rules; Panel **e** – Wavelet principal components (WPCs) resulting from a WPCA using the Daubechies extremal phase mother wavelet and three different shrinkage rules; Panel **f** – Wavelet principal components (WPCs) resulting from a WPCA using the Daubechies least asymmetric mother wavelet and three different shrinkage rules

Results from principal component analysis (PCA) on raw data are shown in Fig. 2a. The first three principal components (PCs) together explained 96.4 % of the total variation between cities. The first PC explained 86.9 % of the total variation and was positively and equally correlated with the load of MDMA on each day of the week. The second PC explained 7.0 % of the total variation and was positively and negatively correlated with the loads on Sunday/Monday and Wednesday/Thursday respectively. The third PC explained 2.4 % of the total variation and was most strongly correlated with the loads on Friday/Saturday.

### Functional principal component analyses

#### Fourier basis functions

For Fourier basis functions using different smoothing parameters, the first three functional principal components (FPCs) are shown in Fig. 2b and supplementary material (Additional file 1: Figure S1 a-c). The first functional principal component (FPC1) explained 88.1-90.8 % of the temporal variation between cities, slightly more than PCA, representing the general level of MDMA in the wastewater. The second FPC (FPC2) explained 6.4-6.9 % of the temporal variation, representing the difference between the midweek level and the weekend peak; cities with a negative FPC2 score had a large difference between the midweek level and the weekend peak of MDMA, with a high level of MDMA at the weekend, while cities with a positive FPC2 score had a small difference between the midweek level and weekend peak of MDMA, with a smoothed load throughout the week. The third FPC (FPC3) explained 2.1-2.6 % of the temporal variation, representing the timing of the weekend peak; cities with a negative FPC3 score had an earlier weekend peak, while cities with a positive FPC3 score had a later weekend peak.

#### B-spline basis functions

For the B-spline basis functions using different smoothing parameters, the first three FPCs are shown in Fig. 2c and supplementary material (Additional file 1: Figure S1 d-f). The first FPC explained 87.5-92.1 % of the observed temporal variation between cities representing the general level of MDMA in the wastewater, while the second and third FPCs explained 5.8-6.8 % and 1.7-2.9 % of the total variation, representing the difference between the midweek level and the weekend peak, and the timing of the weekend peak respectively. The interpretation of the first three FPCs were the same as those for Fourier basis functions, but when using B-splines the third FPC varied quite a lot depending on the choice of the smoothing parameter.

### Wavelet-based principal component analysis

Figure 2d-f show the first three wavelet principal components (WPCs) for each of the three mother wavelets. The temporal patterns are qualitatively consistent with those from PCA and functional principal component analysis (FPCA), but the WPC patterns seem to be somewhat more smoothed throughout the week.

For the Haar wavelet (Fig. 2d), the first three WPCs together explained 94.8-99.6 % of the total variation between cities. The first WPC (WPC1) explained 84.5-87.2 % of the total variation, representing the general level of MDMA in the wastewater. The second WPC (WPC2) explained 7.3-9.2 % of the temporal variation, showing a negative peak on Wednesday/Thursday and a positive peak on Sunday respectively, representing the difference between the midweek level and the weekend peak. Finally the third WPC (WPC3) explained 3.1-3.2 % of the temporal variation, with a peak on Friday/Saturday, representing the timing of the weekend peak.

For Daubechies extremal phase wavelet (Fig. 2e), the first three WPCs explained 96.1-97.9 % of the total variation between cities. WPC1 explained 81.5-82.6 % of the total variation, representing the general level of MDMA in the wastewater. WPC2 explained 12.7-12.8 % of the temporal variation, showing a negative peak on Wednesday and a positive peak on Sunday, representing the difference between the midweek level and the weekend peak. Finally WPC3 explained 1.9-2.4 % of the temporal variation, with a negative peak on Tuesday and a positive peak on Friday/Saturday, representing the timing of the weekend peak.

For Daubechies least asymmetric wavelet (Fig. 2f), the first three WPCs explained 95.9-98.2 % of the total variation between cities. WPC1 explained 83.0-84.4 % of the total variation, representing the general level of MDMA in the wastewater. WPC2 explained 10.1-10.8 % of the temporal variation, with a negative peak on Wednesday and a positive peak on Sunday, representing the difference between the midweek level and the weekend peak. Finally WPC3 explained 2.8-3.0 % of the temporal variation, with negative peaks on Monday/Tuesday and positive peaks on Friday/Saturday, representing the timing of the weekend peak.

### Bootstrapping

**Fig. 3:**
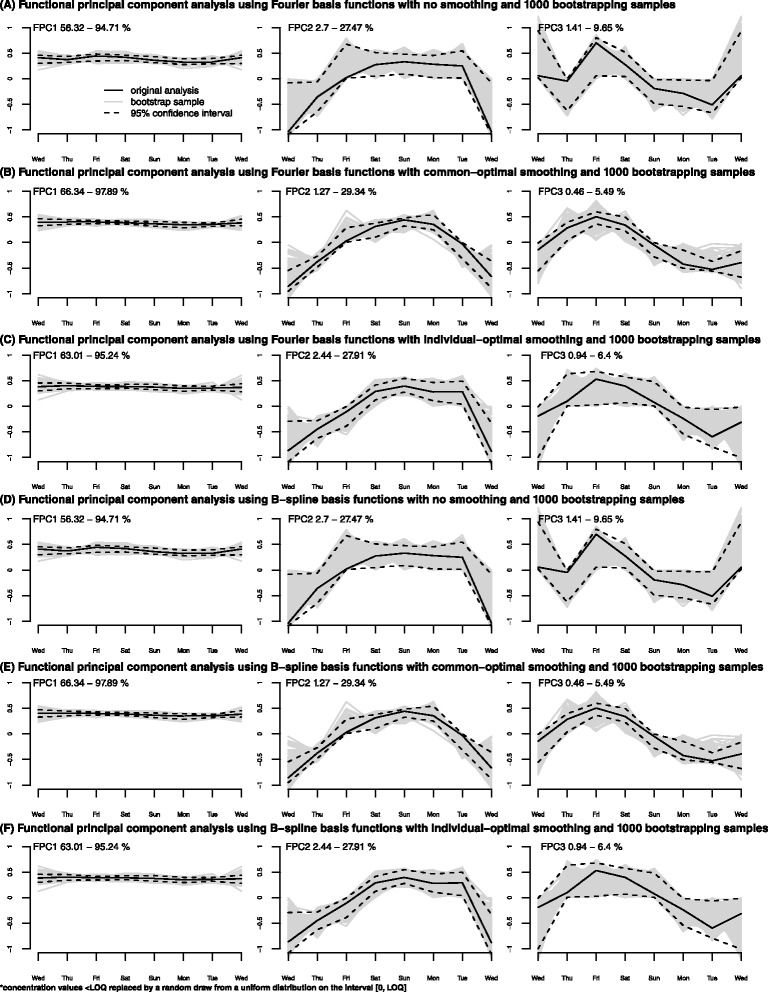
Bootstrapping confidence intervals (CIs) resulting from functional principal component analysis (FPCA) on 1000 re-samples obtained by a random sample with repetition from the original data sets. Panel **a** – Bootstrapping CI resulting from a FPCA using Fourier basis functions and no smoothing parameter; Panel **b** – Bootstrapping CI resulting from a FPCA using Fourier basis functions and common-optimal smoothing parameter; Panel **c** – Bootstrapping CI resulting from a FPCA using Fourier basis functions and individual-optimal smoothing parameter; Panel **d** – Bootstrapping CI resulting from a FPCA using B-splines basis functions and no smoothing parameter; Panel **e** – Bootstrapping CI resulting from a FPCA using B-splines basis functions and common-optimal smoothing parameter; Panel **f** – Bootstrapping CI resulting from a FPCA using B-splines basis functions and individual-optimal smoothing parameter

The bootstrapping confidence intervals (CIs) for Fourier and B-spline basis functions for each FPC and smoothing parameter are shown in Fig. 3.

For the Fourier basis function, the bootstrapping shows that the FPCs are quite stable for each choice of smoothing parameter (Fig. 3a-c). The 95 % CIs are narrower for the first FPC than for the second and third FPCs. The results further indicate that common-optimal smoothing (Fig. 3b) is the best choice when using Fourier basis functions as the FPCs fluctuate less and the CIs are narrower, especially for the third FPC.

For the B-spline basis functions, the bootstrapping indicates that the FPCs are less stable compared to the Fourier basis (Fig. 3d-f). Also, in this case, the 95 % CIs are narrower for the first FPC than for the second and third FPCs. Again common-optimal smoothing (Fig. 3e) appears to be the best choice when using B-spline basis functions as the FPCs fluctuate less and the CIs are narrower, especially for the third FPC.

Results for WPCA were similar to those of FPCA (not shown).

### Sensitivity to missing data

**Fig. 4:**
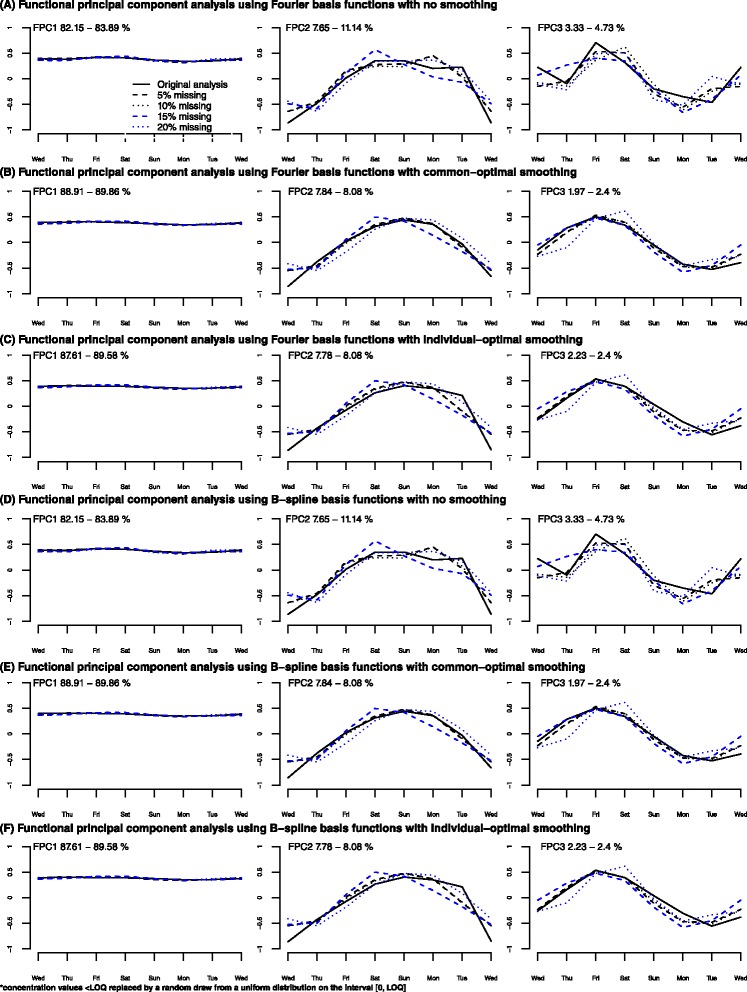
Sensitivity to missing for functional principal component analysis (FPCA) results. Panel **a** – Functional principal components (FPCs) resulting from a FPCA using Fourier basis functions and no smoothing parameter for 5, 10, 15, 20 % of missing; Panel **b** – Functional principal components (FPCs) resulting from a FPCA using Fourier basis functions and common-optimal smoothing parameter for 5, 10, 15, 20 % of missing; Panel **c** – Functional principal components (FPCs) resulting from a FPCA using Fourier basis functions and individual-optimal smoothing parameter for 5, 10, 15, 20 % of missing; Panel **d** – Functional principal components (FPCs) resulting from a FPCA using B-splines basis functions and no smoothing parameter for 5, 10, 15, 20 % of missing; Panel **e** – Functional principal components (FPCs) resulting from a FPCA using B-splines basis functions and common-optimal smoothing parameter for 5, 10, 15, 20 % of missing; Panel **f** – Functional principal components (FPCs) resulting from a FPCA using B-splines basis functions and individual-optimal smoothing parameter for 5, 10, 15, 20 % of missing

For the Fourier basis functions, the analyses indicate that, without smoothing, the FPCs are stable up to 5 % missing data (Fig. 4a), while for individual-optimal smoothing and for the often recommended common smoothing the FPCs are stable up to 15 % missing data (Fig. 4b-c).

For the B-spline basis functions, the analyses indicate that without smoothing the FPCs are not stable even at 5 % missing data (Fig. 4d), while for individual-optimal smoothing and the recommended individual-optimal common smoothing the FPCs are stable up to 10 % missing data (Fig. 4e and f). Considerably more fluctuation is found in each FPC for B-spline basis functions compared to Fourier basis functions.

Results for WPCA were similar to those of FPCA (not shown).

### Values below LOQ

The temporal patterns extracted by FPCA by imputation of the concentration values below the limit of quantification (LOQ) using the fixed value of LOQ/2 or a random draw from a uniform distribution are qualitatively the same, but temporally shifted (not shown). However, the temporal shift in those patterns is balanced by the city scores on each FPC, indicating that the crude imputation by LOQ/2 works reasonably well in practical applications.

## Discussion

In this study, we have explored functional principal component analysis (FPCA) as a tool for analysing temporal wastewater data, comparing it to traditional principal component analysis (PCA) and the temporally more flexible wavelet PCA (WPCA), as well as exploring the robustness of the extracted FPCA patterns and sensitivity to missing data. The results were generally consistent between PCA, FPCA and WPCA. WPCA did not detect any rapid temporal changes in the data. FPCA thus appears not to smooth away essential information in the temporal data, and there is no need to go beyond FPCA to the less tractable WPCA. The analyses establish FPCA using Fourier basis functions and common optimal smoothing as a precise, flexible and stable method for analysing wastewater-based epidemiology (WBE) data.

WBE provides an objective estimate of the use of a specific drug for all people contributing to the wastewater treatment plant in a catchment area over a time period. Recently the advantages of functional data analysis (FDA) over the traditional statistical analyses usually applied to WBE data for information extraction have been demonstrated [18]. FDA is analytically tractable and a well-established mathematical framework for temporal data [25], and a series of R packages for calculations exist [26]. This greatly assists the introduction of more advanced statistical analysis to a novel field within the health sciences, and the initial concern that FDA might over-smooth the underlying temporal process in wastewater data was shown to be non-existent. FDA is indeed sufficiently flexible and stable for the analysis of WBE data.

In this work, we have investigated FDA for WBE further. First comparing various FPCA using both Fourier and B-spline basis functions [25, 26] to crude PCA [24] and the more temporally flexible WPCA [22, 23]. We further explored the stability of the FPCA results through bootstrapping, and sensitivity to missing data by randomly deleting 5, 10, 15 and 20 % of the original data. FPCA using Fourier basis functions stood out as the most accurate method. Performing the same analyses for WPCA gave similar results, but as WPCA was found to not add any new insight beyond FPCA, results from these further analyses are not presented.

A Fourier basis is particularly useful for periodic data, where the temporal pattern is stable, there are no strong local features and the curvature of the underlying process tends to be of the same order everywhere [25]. The single week time period often found in WBE data, and investigated in this work, lies on the boundary between a periodic and non-periodic temporal process. In the latter scenario, B-spline basis functions tend to perform better [25], combining polynomials with greater flexibility.

Using Fourier basis functions, the patterns shown by each functional principal component (FPC) are consistent regardless of the choice of smoothing. A common optimal smoothing parameter did, however, lead to an increase in the total variation explained by the first FPC (FPC1). Using B-splines temporal patterns were mainly the same as those found using a Fourier basis. However, the third FPC appears less capable of modelling the difference between weekdays and weekend loads, and there were larger differences between the different choices of smoothing parameter. Overall common-optimal smoothing seemed to perform better than ‘no smoothing’ or individual-optimal smoothing, where some spurious variability was detected.

Naïve statistical analyses have pointed to a significant difference in wastewater drug loads between weekdays and the weekend [15–17]. FDA does however indicate a smooth transition between weekdays and the weekend [18], blurring the lines between which days actually constitute the weekend. It was, therefore, a concern that the FDA approach might over-smooth the data, so we explored WPCA as an alternative. Wavelet basis functions, unlike Fourier and B-spline basis functions, are able to model more extreme behaviour within a temporal phenomenon, even spikes [22, 23]. They work well with discontinuity or rapid changes, combining the frequency-domain of the Fourier series with the time-localized features of splines [21], and should thus be able to model a more rapid change between weekdays and weekend, should this rapid change indeed exist.

While wavelets are flexible, wavelet basis functions are not expressed explicitly as functions, with individual basis functions specified by recursive difference equations conditioned on a mother wavelet, making the wavelet basis system less tractable than Fourier or B-spline bases [22].

The temporal features extracted by wavelet principal components (WPCs) using the Haar wavelet indicate a principal difference between weekdays and weekend loads. Indeed, the analysis almost points to a possible dichotomization of the data in this sense. While this would be in line with our society’s cultural a priori definition of weekdays and weekend, the day-by-day results as found by crude PCA indicates that this might be an oversimplification. All analyses other than Haar wavelets indicate a smooth transition between weekday and weekend loads.

Results using Daubechies’ wavelets are similar to those found by traditional PCA on the raw data, except the first WPC explained more of the temporal variation from the ecstasy (MDMA) curves. Generally, in the WPCA the choice of the thresholding rule when performing the wavelet shrinkage had relatively little impact on the WPCA results.

Even though the patterns extracted by PCA, FPCA and WPCA were qualitatively consistent, the interpretation of the principal components (PCs) and WPCs can be difficult to compare to the FPCs. In PCA, individual days are assumed to be independent variables, that is, the drug load at a given day is independent of the drug load at any other day, so PCA does not take the possible correlation between consecutive days of the temporal data set into account. PCA on WBE is generally not advised, since it treats days as independent entities without controlling for the intra-correlation between them, ignoring the fact that the seven days constitute a single entity, a week. Further, WPCA is an extension of traditional PCA, but less direct than FPCA. In the version of WPCA applied here, PCA is performed on the smoothed wavelet coefficients in wavelet domain, where the wavelet coefficients constitute the independent variables for the subsequent PCA, before back calculating each WPC to time domain. As a result the patterns of the WPCs do not have the same scale as the PCs and FPCs, making direct comparison between the methods difficult.

The WPCA is not only less mathematically tractable than the FPCA approach. The epidemiological interpretation of the FPCs is often easier as the FPC curves can be illustrated by plots showing how an individual curve differs from the mean curve if the FPC scores are high or low (Additional file 1: Figure S1), rather than mere plotting of the FPC curves [26].

When exploring FPCA further, the bootstrapping showed that, using Fourier basis, the confidence intervals (CIs) are narrower than all the other cases, and when exploring sensitivity to missing data we found that results are stable even with 15 % missing data.

Both this and earlier explorations of FDA for WBE data are restricted by having only one week of observations. Longer observation times would allow for statistical methods that could separate long-term temporal changes from the weekly pattern, such as the Fourier Poisson time-series regression model recently applied to suicide counts [42]. Similarly, more frequent sampling would allow for assessing changes throughout both day and week.

## Conclusions

In this paper we have explored FPCA as a method for analysing temporal wastewater data. FPCA extracted all temporal features discovered by the temporally more flexible wavelet approach, overcoming the initial concern of over-smoothing the underlying temporal process. The FPCA approach was not particularly sensitive to the choice of basis function or to missing data, but Fourier basis functions with common-optimal smoothing parameter generally performed better.

## Additional file

Additional file 1:
**Figure S1.** Functional principal components (FPCs) resulting from functional principal component analysis (FPCA). Shows the mean of the fitted curves (solid line) and how the shape of an individual curve differs from the mean curve if a multiple of the principal component curve is added to (+ +) or subtracted from (- -) the mean curve. Panel A –First three FPCs resulting from a FPCA using Fourier basis functions and no smoothing parameter; Panel B – First three FPCs resulting from a FPCA using Fourier basis functions and common-optimal smoothing parameter; Panel C – First three FPCs resulting from a FPCA using Fourier basis functions and individual-optimal smoothing parameter; Panel D – First three FPCs resulting from a FPCA using B-splines basis functions and no smoothing parameter; Panel E – First three FPCs resulting from a FPCA using B-splines basis functions and common-optimal smoothing parameter; Panel F – First three FPCs resulting from a FPCA using B-splines basis functions and individual-optimal smoothing parameter. (PDF 27 kb)
