# Erratum to: Exploring functional data analysis and wavelet principal component analysis on ecstasy (MDMA) wastewater data

**DOI:** 10.1186/s12874-017-0311-y

**Published:** 2017-02-23

**Authors:** Stefania Salvatore, Jørgen G. Bramness, Jo Røislien

**Affiliations:** 1 Norwegian Centre for Addiction Research, University of Oslo, Oslo, Norway; 2 Oslo Centre for Biostatistics and Epidemiology, Institute of Basic Medical Sciences, Oslo, Norway

## Erratum

After publication of the original article [1], it came to the authors’ attention that there were errors in Fig. 3, Fig. 4 and Additional file 1: Figure S1.

**Fig. 3:**
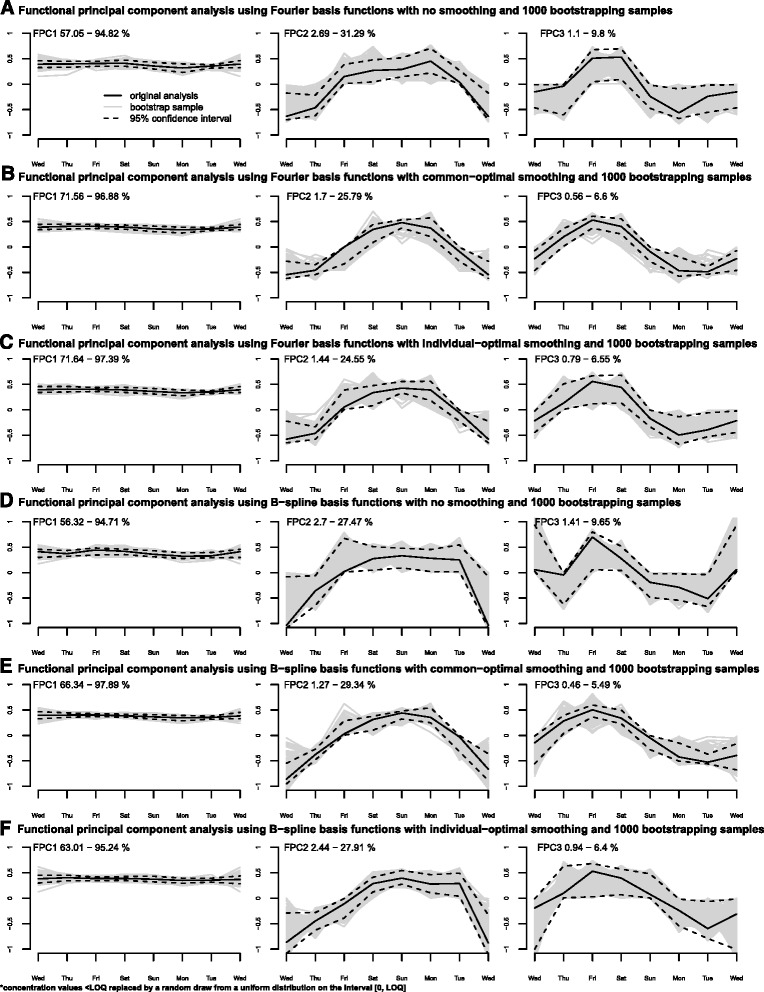
Bootstrapping confidence intervals (CIs) resulting from functional principal component analysis (FPCA) on 1000 re-samples obtained by a random sample with repetition from the original data sets. Panel **a** – Bootstrapping CI resulting from a FPCA using Fourier basis functions and no smoothing parameter; Panel **b** – Bootstrapping CI resulting from a FPCA using Fourier basis functions and common-optimal smoothing parameter; Panel **c** – Bootstrapping CI resulting from a FPCA using Fourier basis functions and individual-optimal smoothing parameter; Panel **d** – Bootstrapping CI resulting from a FPCA using B-splines basis functions and no smoothing parameter; Panel** e** – Bootstrapping CI resulting from a FPCA using B-splines basis functions and common-optimal smoothing parameter; Panel** f** – Bootstrapping CI resulting from a FPCA using B-splines basis functions and individual-optimal smoothing parameter

**Fig. 4:**
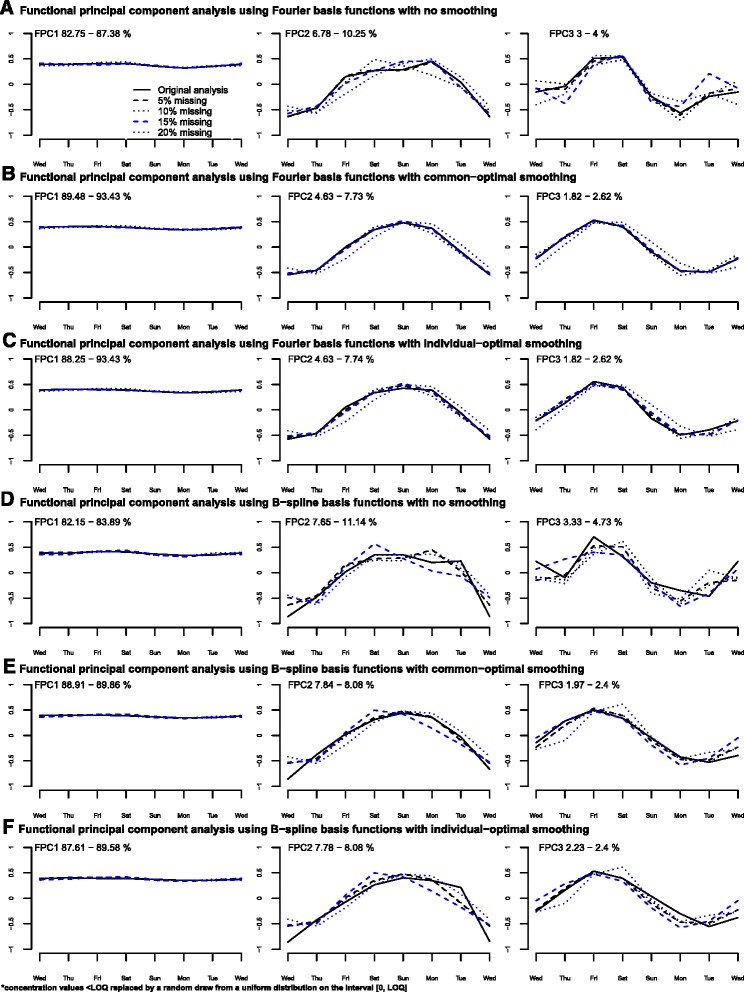
Sensitivity to missing for functional principal component analysis (FPCA) results. Panel **a** – Functional principal components (FPCs) resulting from a FPCA using Fourier basis functions and no smoothing parameter for 5, 10, 15, 20 % of missing; Panel **b** – Functional principal components (FPCs) resulting from a FPCA using Fourier basis functions and common-optimal smoothing parameter for 5, 10, 15, 20 % of missing; Panel **c** – Functional principal components (FPCs) resulting from a FPCA using Fourier basis functions and individual-optimal smoothing parameter for 5, 10, 15, 20 % of missing; Panel **d** – Functional principal components (FPCs) resulting from a FPCA using B-splines basis functions and no smoothing parameter for 5, 10, 15, 20 % of missing; Panel **e** – Functional principal components (FPCs) resulting from a FPCA using B-splines basis functions and common-optimal smoothing parameter for 5, 10, 15, 20 % of missing; Panel **f** – Functional principal components (FPCs) resulting from a FPCA using B-splines basis functions and individual-optimal smoothing parameter for 5, 10, 15, 20 % of missing

In each Figure, panels A, B and C are not correct (but panels D, E and F are). This error was due to a mistake in the last stages of the submission process while adjusting the Figures’ size to fit the journal’s requirements. This error does not impact the results, discussion and conclusions of the paper.

The correct version of the affected Figures are published in this erratum.

## **Additional file**

Additional file 1: Figure S1. (A) Functional principal component analysis using Fourier basis functions with no smoothing. (B) Functional principal component analysis using Fourier basis functions with common−optimal smoothing. (C) Functional principal component analysis using Fourier basis functions with individual−optimal smoothing. (D) Functional principal component analysis using B−spline basis functions with no smoothing. (E) Functional principal component analysis using B−spline basis functions with common−optimal smoothing. (F) Functional principal component analysis using B−spline basis functions with individual−optimal smoothing. (PDF 27 kb)
