# Supplementary material for: Exploring functional data analysis and wavelet principal component analysis on ecstasy (MDMA) wastewater data
Source: BMC Med Res Methodol. 2016 Jul 12;16:81. doi: 10.1186/s12874-016-0179-2 (PMC4942983; doi:10.1186/s12874-016-0179-2)
Supplement: Additional file 1: — Figure S1. Functional principal components (FPCs) resulting from functional principal component analysis (FPCA). Shows the mean of the fitted curves (solid line) and how the shape of an individual curve differs from the mean curve if a multiple of the principal component curve is added to (+ +) or subtracted from (- -) the mean curve. Panel A –First three FPCs resulting from a FPCA using Fourier basis functions and no smoothing parameter; Panel B – First three FPCs resulting from a FPCA using Fourier basis functions and common-optimal smoothing parameter; Panel C – First three FPCs resulting from a FPCA using Fourier basis functions and individual-optimal smoothing parameter; Panel D – First three FPCs resulting from a FPCA using B-splines basis functions and no smoothing parameter; Panel E – First three FPCs resulting from a FPCA using B-splines basis functions and common-optimal smoothing parameter; Panel F – First three FPCs resulting from a FPCA using B-splines basis functions and individual-optimal smoothing parameter. (PDF 27 kb) [file 12874_2016_179_MOESM1_ESM.pdf]

(A) Functional principal component analysis using Fourier basis functions with no smoothing

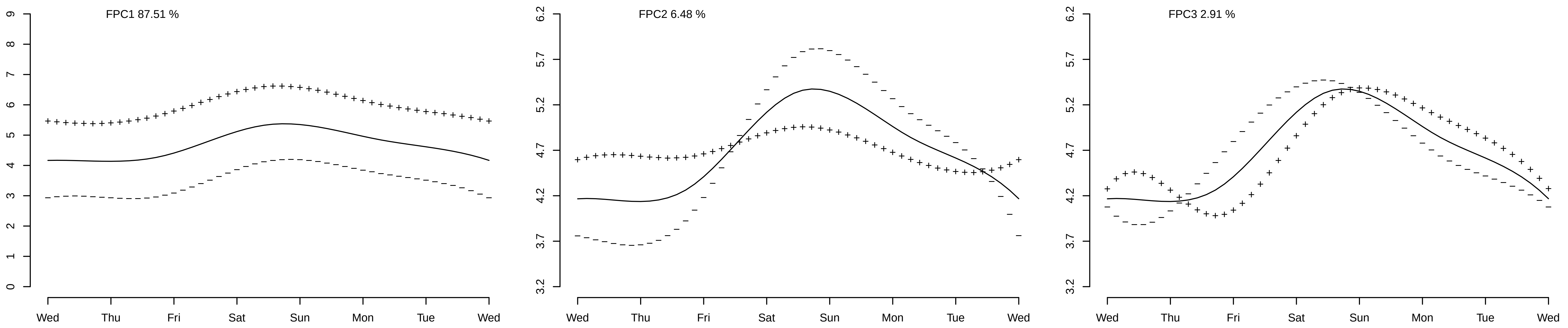

(B) Functional principal component analysis using Fourier basis functions with common−optimal smoothing

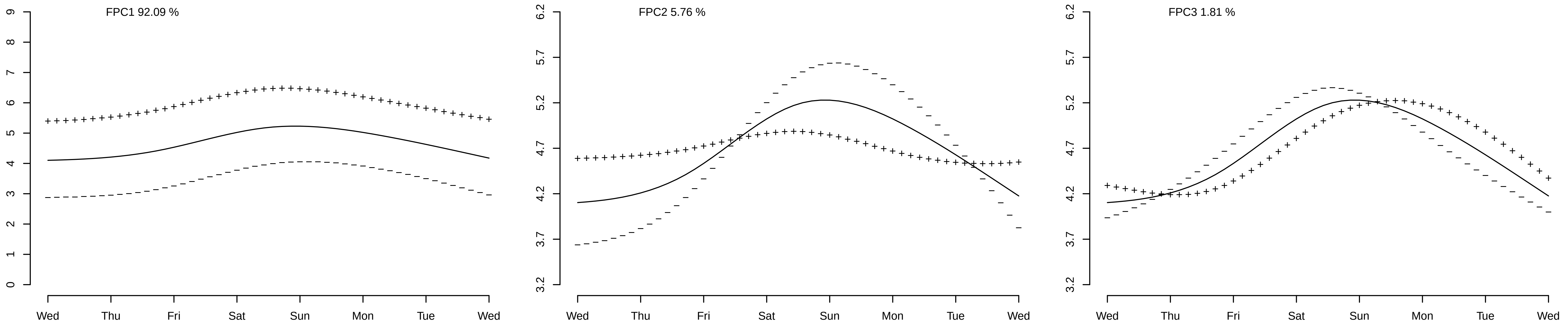

(C) Functional principal component analysis using Fourier basis functions with individual−optimal smoothing

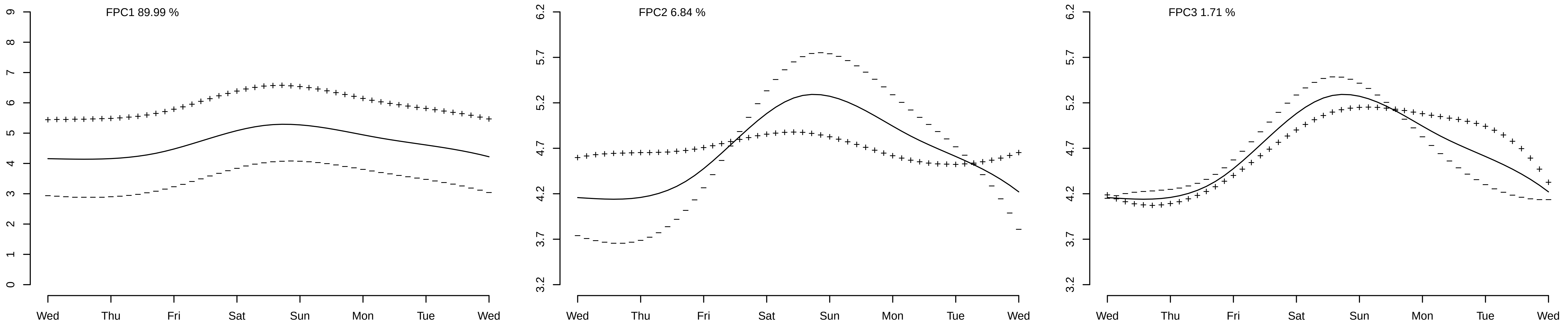

(D) Functional principal component analysis using B−spline basis functions with no smoothing

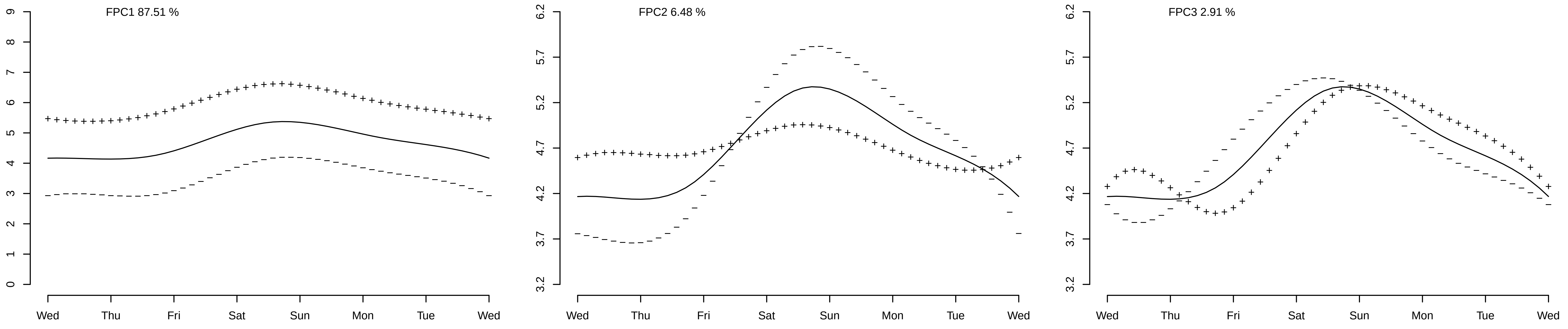

(E) Functional principal component analysis using B−spline basis functions with common−optimal smoothing

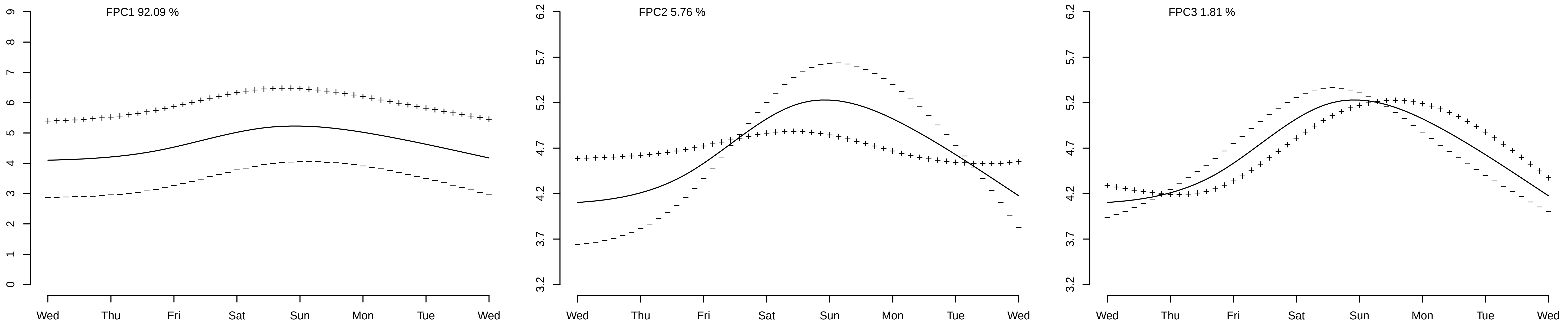

(F) Functional principal component analysis using B−spline basis functions with individual−optimal smoothing

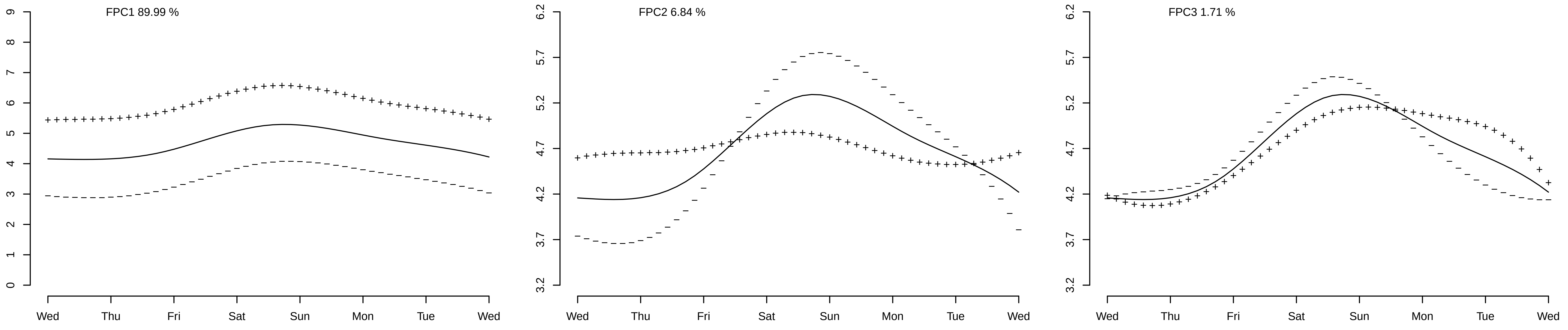

***concentration values <LOQ replaced by a random draw from a uniform distribution on the interval [0, LOQ]**
