# Supplementary material for: Erratum to: Exploring functional data analysis and wavelet principal component analysis on ecstasy (MDMA) wastewater data
Source: BMC Med Res Methodol. 2017 Feb 23;17:34. doi: 10.1186/s12874-017-0311-y (PMC5324302; doi:10.1186/s12874-017-0311-y)
Supplement: Additional file 1: Figure S1. — (A) Functional principal component analysis using Fourier basis functions with no smoothing. (B) Functional principal component analysis using Fourier basis functions with common−optimal smoothing. (C) Functional principal component analysis using Fourier basis functions with individual−optimal smoothing. (D) Functional principal component analysis using B−spline basis functions with no smoothing. (E) Functional principal component analysis using B−spline basis functions with common−optimal smoothing. (F) Functional principal component analysis using B−spline basis functions with individual−optimal smoothing. (PDF 27 kb) [file 12874_2017_311_MOESM1_ESM.pdf]

S1 Figure

(A) Functional principal component analysis using Fourier basis functions with no smoothing

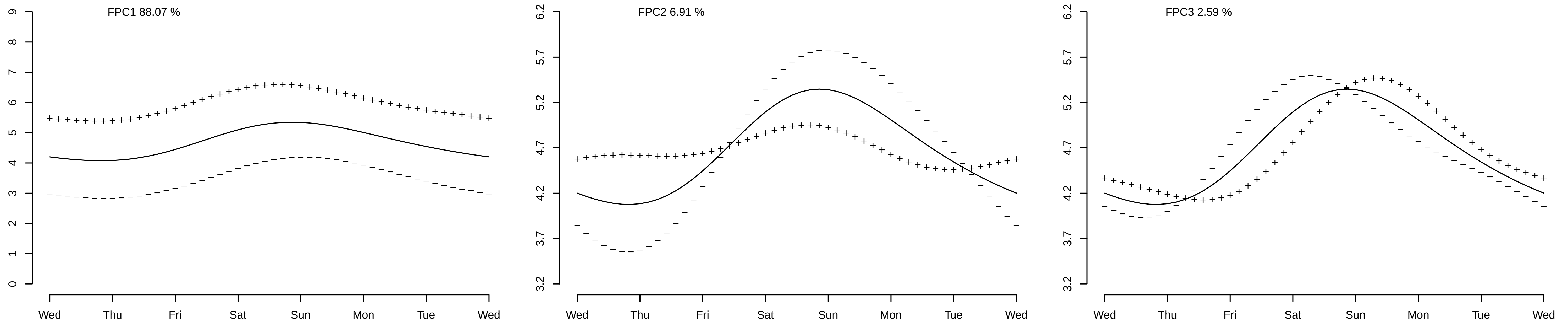

(B) Functional principal component analysis using Fourier basis functions with common−optimal smoothing

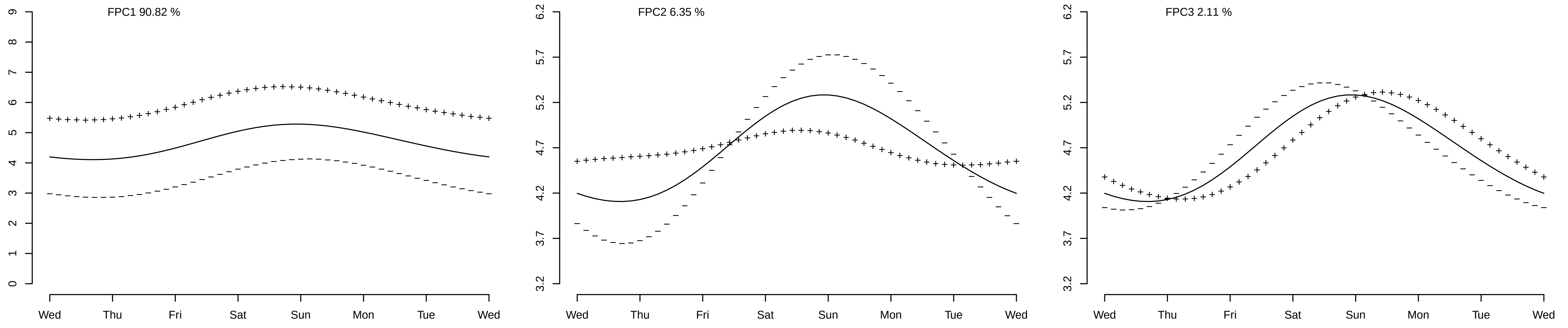

(C) Functional principal component analysis using Fourier basis functions with individual−optimal smoothing

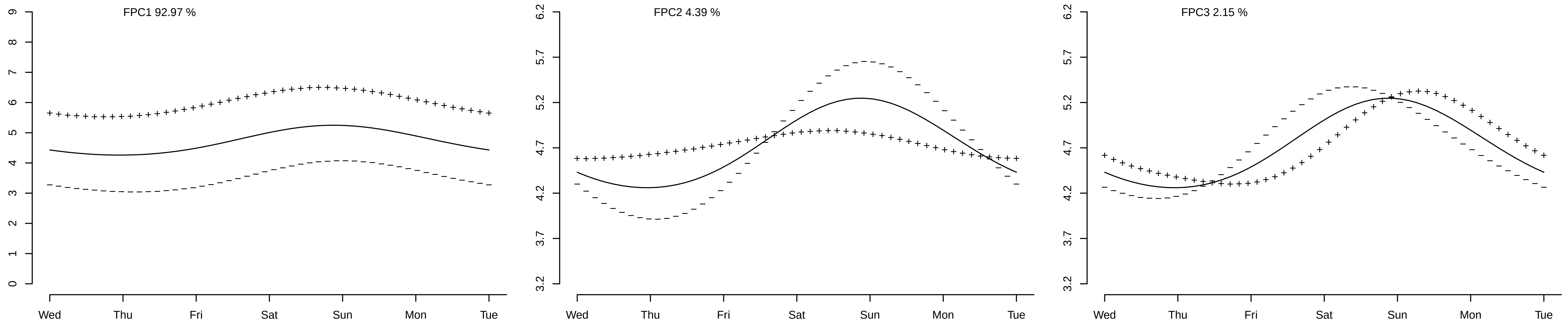

(D) Functional principal component analysis using B−spline basis functions with no smoothing

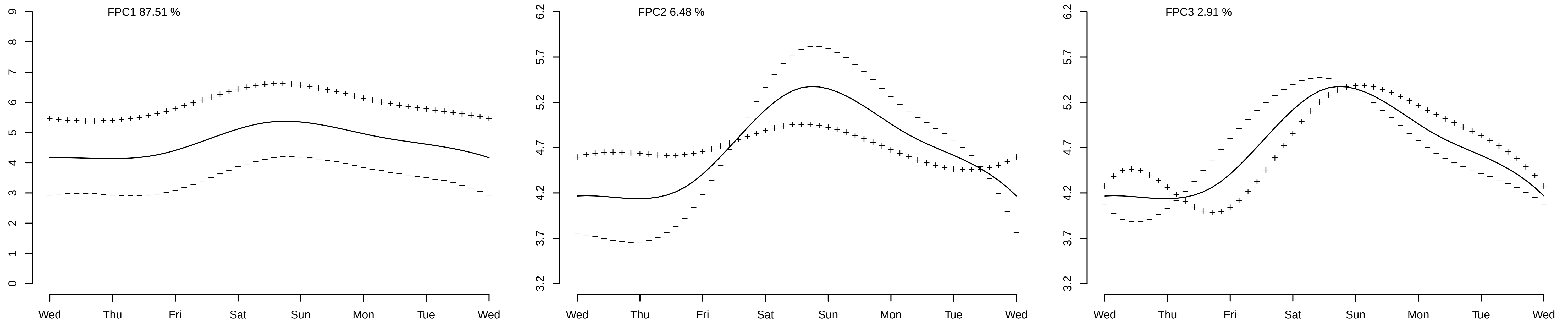

(E) Functional principal component analysis using B−spline basis functions with common−optimal smoothing

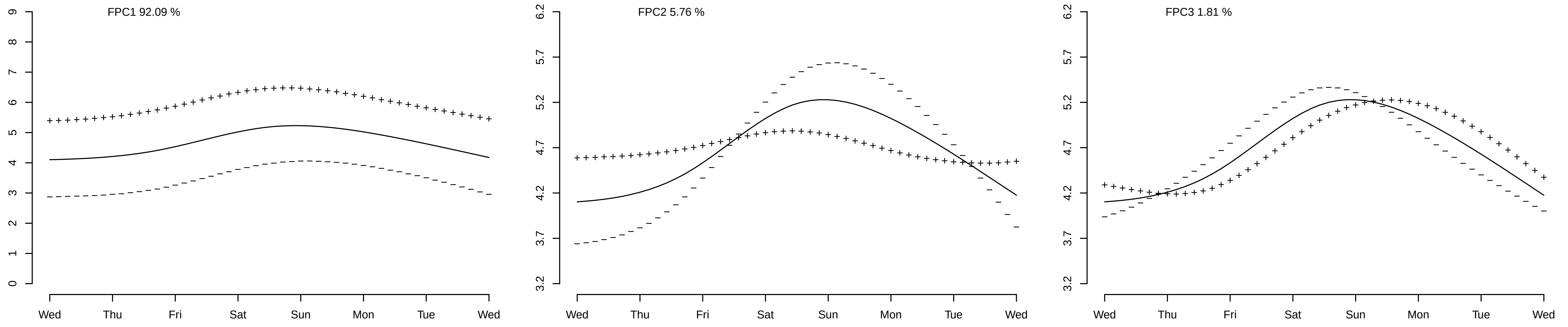

(F) Functional principal component analysis using B−spline basis functions with individual−optimal smoothing

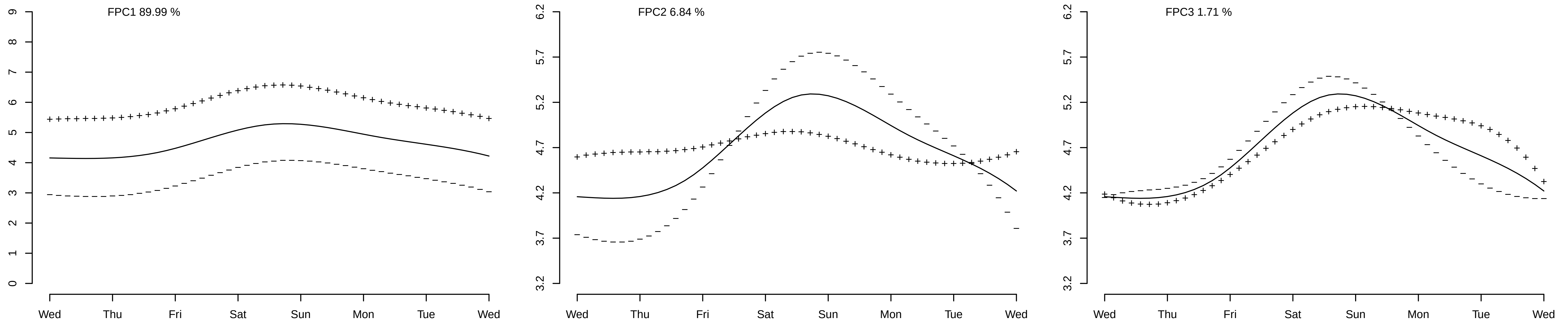

*concentration values <LOQ replaced by a random draw from a uniform distribution on the interval [0, LOQ]
